# Protonated Organic Diamines as Templates for Layered and Microporous Structures: Synthesis, Crystal Chemistry, and Structural Trends among the Compounds Formed in Aqueous Systems Transition Metal Halide or Nitrate–Diamine–Selenious Acid

**DOI:** 10.3390/ijms241814202

**Published:** 2023-09-17

**Authors:** Dmitri O. Charkin, Evgeny V. Nazarchuk, Dmitri N. Dmitriev, Vasili Yu. Grishaev, Timofey A. Omelchenko, Darya V. Spiridonova, Oleg I. Siidra

**Affiliations:** 1 Inorganic Chemistry Division, Chemistry Department, Moscow State University, Vorobievy Gory, 1-3, 199991 Moscow, Russia; d.o.charkin@gmail.com (D.O.C.); ddn063@gmail.com (D.N.D.); tomelchenko003@gmail.com (T.A.O.); 2 Department of Crystallography, Institute of Earth Sciences, St. Petersburg State University, 199034 St. Petersburg, Russia; e_nazarchuk@mail.ru (E.V.N.); grishaevv98@mail.ru (V.Y.G.); 3 X-ray Diffraction Resource Center, St. Petersburg State University, 199034 St. Petersburg, Russia; daria.spiridonova@spbu.ru; 4 Kola Science Center, Russian Academy of Sciences, Fersman Str. 14, 184209 Apatity, Russia

**Keywords:** selenites, transition metals, metal halides, organically templated compounds, synthesis, microporous structures, protonated organics

## Abstract

Systematic studies of crystalline compounds formed in aqueous systems containing aliphatic diamines, divalent transition metal halides, and selenious acid resulted in the discovery of a large family of new complex species corresponding to several new structure types. With ethylenediamine (en), layered (enH_2_)[*M*(HSeO_3_)_2_*X*_2_] compounds are the most commonly formed species which constitute a significant contribution to the family of layered hydrogen selenites containing neutral [*M*(HSeO_3_)_2_] (*M* = Mg, Mn, Co, Ni, Cu, Zn, Cd) 2*D* building blocks. In contrast to some previous suggestions, piperazine (pip), as well as its homologue N-methylpiperazine, mostly give rise to quite different, sometimes more complex, structures of varied dimensionality while the (pipH_2_)[*M*(HSeO_3_)_2_*X*_2_] compounds are formed only with *M* = Cu and Cd. In addition, metal-, halide-, or selenium-free by-product species are observed. The Se^IV^ can be present in a multitude of forms, including H_2_SeO_3_, HSeO_3_^−^, SeO_3_^2−^, and Se_2_O_5_^2−^, reflecting amazing adaptability to the shape of the templating cations.

## 1. Introduction

The use of “lone-pair” cations and halide or nitrate anions is well known to lead to numerous open-framework, porous, and non-centrosymmetric structures [1,2,3,4,5]; addition of magnetically active cations of *d*- and *f*-elements frequently gives rise to unusual architectures and magnetic responses. Yet, most attention is dedicated to fully deprotonated acid residues, e.g., selenites, tellurites, or iodates, while the acid salts and other hydrogen-bonded networks have as yet received less attention, although at least some of these species exhibit several attractive properties [6,7,8].

A numerous yet sparsely addressed family of mostly inorganic compounds is formed by the “layered hydroselenites”, first described by Trombe et al. [9,10,11] and further extended in our studies [12,13,14,15] among compounds of copper. Later, some zinc [16], cobalt [17,18,19,20], and cadmium [21] containing species were reported. The crystal chemistry of this family is relatively diverse and the overwhelming majority of structure types and their representatives are the copper compounds. The structures of these compounds contain quasi-planar [*M*^II^(HSeO_3_)_2_]^0^ layers formed by Cu^2+^ cations and hydrogen-bonded dimers of HSeO_3_^−^ anions forming a distorted quasi-square grid. The interlayer space can be either empty, as in Cu(HSeO_3_)_2_ [22], filled with water molecules, as in Cu(HSeO_3_)_2_·2H_2_O [9], various metal-halide [11,12,13,15,16,17,20] and metal-nitrate [14] slabs, or more complex species such as [*M*^II^(H_2_O)_4_][Cu(HSeO_3_)_2_Cl_2_] series (*M*^II^ = Mn–Zn, Fe excepted) [10,18] and [Co(H_2_O)_4_][Co(HSeO_3_)_2_Cl_2_] [20]. It is noteworthy that the bromide analogs of the two latter compounds, as well as alkali–cobalt selenite chlorides [17], have not been reported, while within the (*A*^I^*X*)[Cu(HSeO_3_)_2_] series (*A*^I^ = Na, K, Rb, Cs, NH_4_) [13], as well as (Rb*X*)[Zn(HSeO_3_)_2_] [16], bromides are full structural analogs of the corresponding chlorides. Several nitrate-containing copper compounds are also known [9,16].

**Figure 1:**
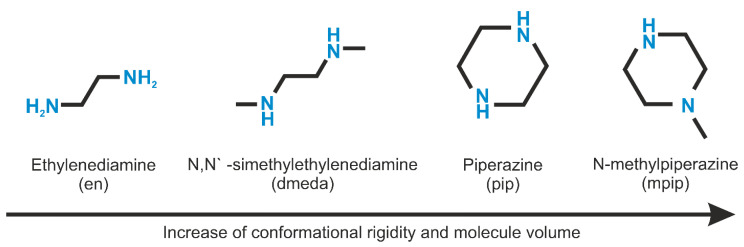
Organic templates used in the current study.

Among halides, ammonium compounds are complete structural analogs of the rubidium- and cesium-containing species [13,16], while among nitrates, they contribute to the compound with the most complex interlayer architecture, (NH_4_NO_3_)_3_[Cu(HSeO_3_)_2_] [9], which was tentatively attributed to the ability of the NH_4_^+^ cations to form strong directional hydrogen bonds to the nitrate anions. This suggests that some other species, particularly organic ammonium cations, may also be incorporated into the interlayer space. This was initially demonstrated by the preparation of cadmium-containing compound (enH_2_)[Cd(HSeO_3_)_2_Cl_2_] (enH_2_^2+^ = ethylenediammonium cation, H_3_NCH_2_CH_2_NH_3_^2+^) [21]; later, isostructural cobalt and copper-based compounds were reported [19]. The layer topology in their structure is essentially the same as that in [*M*(H_2_O)_4_][Cu(HSeO_3_)_2_Cl_2_], [Co(H_2_O)_4_][Co(HSeO_3_)_2_Cl_2_], and [*A*_2_(H_2_O)*_n_*][Co(HSeO_3_)_2_Cl_2_] (*A* = K, *n* = 2; *A* = Cs, *n* = 0). The existence of a related piperazinium compound was also noted in [21]; however, no data have been reported nor possible analogs mentioned. Yet, ethylenediammonium and piperazinium cations are characterized by nearly the same distances between the ammonium hydrogen bond donor centers, though the number of “active” N–H bonds is different (6 vs. 4). In the current study, we performed an extensive search for possible analogs among hydrogen selenite–halides of diprotonated ethylenediamine (enH_2_^2+^), piperazine (pipH_2_^2+^), *N*,*N*′-dimethlethylenediamine (dmedaH_2_^2+^), and N-methylpiperazine (mpipH_2_^2+^) species (Figure 1) and transition metals reported earlier to form the [*M*^II^(HSeO_3_)_2_] slabs, namely Co, Cu, Zn, and Cd. In addition, test experiments were also performed with magnesium, manganese, and nickel halides.

The outcomes of the solution syntheses depended mostly on the nature of the organic species used. The majority of the new species belong to the targeted hydroselenite family; however, representatives of other, more compositionally simple families were also observed. These can be roughly collected into the following groups: (i) the “layered hydroselenites” containing all elements involved; (ii) framework selenite–diselenites with no incorporated halide or nitrate; (iii) ion-molecular crystals of ethylenediammonium salts and selenious acid which do not contain the divalent metal; and (iv) halometallates of organic cations which do not incorporate selenium. 

## 2. Results and Discussion

### 2.1. (enH_2_)[M(HSeO_3_)_2_X_2_] (X = Cl and Br)

Representatives of this structure type have been found among compounds of Co, Cu, Zn, and Cd, that is, for all *M*^2+^ cations where the formation of [*M*(HSeO_3_)_2_] layers had been reported (vide supra); the new contributors to this series are Mn^2+^, Ni^2+^, and probably Mg^2+^ (Table 1). Similar to (*AX*)[*M*(HSeO_3_)_2_] (*M* = Cu, Zn) and in contrast to [*M*′(H_2_O)_4_][*M*(HSeO_3_)_2_], both chlorides and bromides were found to exist. Bromides have not been observed yet for Mg^2+^ and Ni^2+^; most likely these compounds exist but are less stable and more sensitive to the preparation conditions. Numerous attempts to prepare acceptable quality crystals for the elusive (enH_2_)[Mg(HSeO_3_)_2_Cl_2_] have not been successful; the cell metrics and positions of non-hydrogen atoms correspond to the same arrangement.

**Figure 2:**
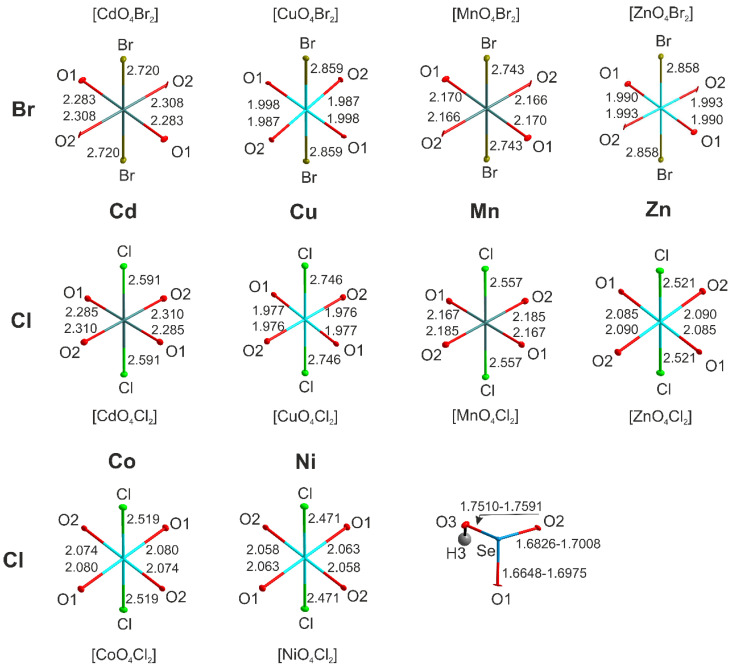
Cation coordination in (enH_2_)[*M*(HSeO_3_)_2_*X*_2_].

In the structures of (enH_2_)[*M*(HSeO_3_)_2_*X*_2_] (*M* = Cd, Co, Cu, Mn, Zn; *X* = Cl, Br), the metal cations center slightly distorted *trans*-*M*O_4_*X*_2_ octahedra (Figure 2). The bond valence sums on *M*^2+^ cites agree well with the oxidation state of 2. The distortion indices for the octahedra (Table 2) were calculated using the Vesta suite [23].

The largest anisotropy in the bond lengths is observed among the Cu and Zn compounds. The difference between the apical and equatorial bonds in [CuO_4_Br_2_] is as much as 0.871Å (distortion index = 0.168). In the most symmetrical [CdO_4_Cl_2_] octahedron, this difference drops to 0.293 Å (distortion index = 0.055). The strong distortion of the Cu and Zn polyhedral can be explained considering the low CFSE values and the Jahn–Teller effect (first order for the former and second order for the latter). Another reason for the distortion of the *M*O_4_*X*_2_ octahedra is extrinsic and caused by hydrogen bonds, both to oxygen and halogen vertices, from the (enH_2_)^2+^ or (pipH_2_)^2+^ templates, where the size of halogen also matters. The volumes of the *M*O_4_*X*_2_ octahedra vary from 14.27 Å^3^ (CuO_4_Cl_2_) to 19.06 Å^3^ (CdO_4_Br_2_). The *M*-*X* bond distances agree well with the reported values, e.g., Cd-Br distances of 2.7198(3) Å are only slightly shorter than those in the CdBr_6_ octahedra in CdBr_2_ (2.785(4)Å); the same applies to the Cd–Cl bond distances in (enH_2_)[Cd(HSeO_3_)_2_Cl_2_] (2.5910(7) Å) and CdCl_2_ (2.637(4)Å). Selenium forms the expected SeO_3_ ψ-tetrahedron with two shorter (1.67–1.69Å) and one longer (1.75–1.77 Å) bond to the OH group (Figure 2) as expected for the HSeO_3_^−^ anion. 

**Figure 3:**
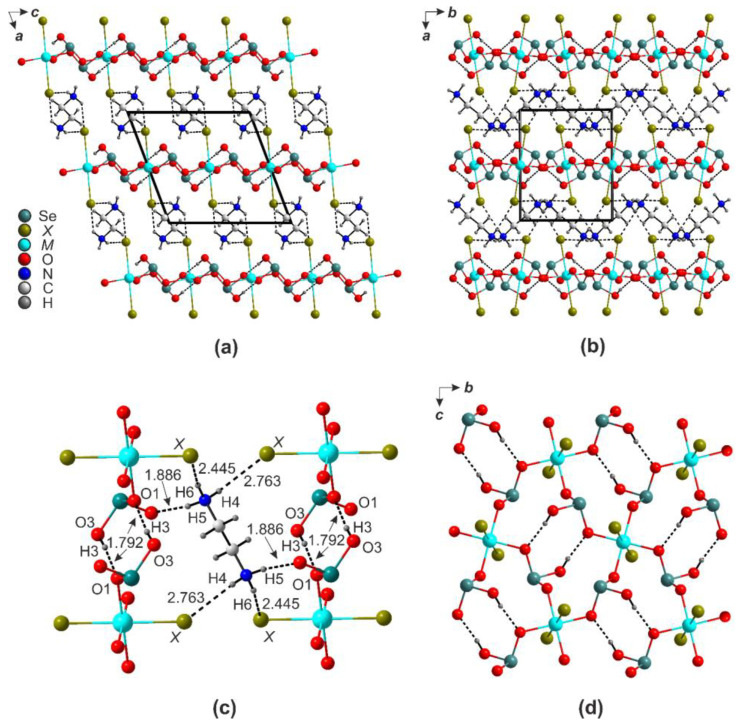
Projection of the structures of (enH_2_)[*M*(HSeO_3_)_2_*X*_2_] onto *ac* (**a**) and *ab* (**b**) planes. Hydrogen bonding systems: enH_2_-*X*-enH_2_ (**c**) and HSeO_3_-HSeO_3_ (**d**).

A typical crystal structure of the (enH_2_)[*M*(HSeO_3_)_2_*X*_2_] (*M* = Cd, Co, Cu, Mn, Zn, Ni; *X* = Cl, Br) is shown in Figure 3a,b. The *M*^2+^ cations reside in centers of *M*O_4_*X*_2_ octahedra formed by the non-protonated oxygen atoms of the four HSeO_3_^−^ anions; these are very similar to those in the corresponding chloride compound (2.2881(17) and 2.3082(18) Å, respectively [21]). Each of the protonated nitrogen atoms of the enH_2_^2+^ cation forms hydrogen bonds: two to the bromide anion and one to the oxygen atoms of two HSeO_3_^−^ species (Figure 3c). The halide anions’ hydrogen bonds are weakest (3.196(5)–3.435(3)Å). Therefore, the enH_2_^2+^ cation forms the maximal number (6) of possible hydrogen bonds. Very similar environments are also observed in the compounds of manganese, cobalt, nickel, copper, and zinc.

The [*M*O_4_*X*_2_] octahedra are stitched by the hydrogen-bonded (HSeO_3_^−^)_2_ pairs into the [*M*(HSeO_3_)_2_*X*_2_]^2−^ layers (Figure 3d). In the structures of (enH_2_)[*M*(HSeO_3_)_2_*X*_2_], the O3-H1···O1 bond lengths (Table S1) change within 2.616(2)–2.653(4) Å, while the angles change within 158.11–175.90°. The enH_2_^2+^-HSeO_3_^−^ hydrogen bonds are rather insensitive to the nature of *M*^2+^ cations (2.798(2)–2.971(1)Å). 

### 2.2. The (pipH_2_)[Cd(HSeO_3_)_2_X_2_] (X = Cl, Br) Series

In proper agreement with [21], two isostructural (pipH_2_)[Cd(HSeO_3_)_2_*X*_2_] compounds were prepared (Table 3). 

**Figure 4:**
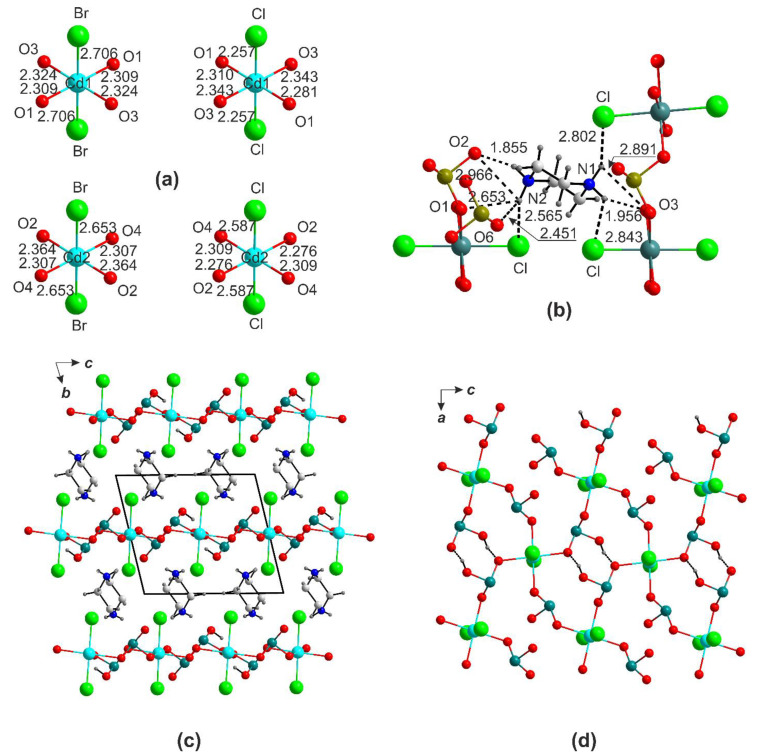
Cation coordination in the crystal structure of (pipH_2_)[Cd(HSeO_3_)_2_*X*_2_] (**a**). Hydrogen bonding systems: pipH_2_-Br (**b**). Projection of the structures of (pipH_2_)[Cd(HSeO_3_)_2_*X*_2_] onto *ab* plane (**c**). The [Cd(HSeO_3_)_2_*X*_2_]^2−^ layer in the structure of (pipH_2_)[Cd(HSeO_3_)_2_*X*_2_] (**d**).

In the structures of (pipH_2_)[Cd(HSeO_3_)_2_*X*_2_], the Cd atoms also center the [CdO_4_*X*_2_] octahedra (Figure 4a). The mean Cd-O separations in the [CdO_4_Br_2_] (2.335(3) Å) and [CdO_4_Cl_2_] (2.293(4) Å) in (enH_2_)[*M*(HSeO_3_)_2_*X*_2_] and (pipH_2_)[Cd(HSeO_3_)_2_*X*_2_] are rather close, yet Cd-*X* (<Cd-Br> = 2.653 Å, <Cd-Cl> = 2.587 Å). 

The bond differences in the CdO_4_*X*_2_ octahedra (0.318 and 0.577 Å for [CdO_4_Br_2_] and [CdO_4_Cl_2_], respectively) are smaller in (pipH_2_)[Cd(HSeO_3_)_2_*X*_2_] compared to (enH_2_)[Cd(HSeO_3_)_2_*X*_2_]. The piperazinium cations form five hydrogen bonds of varying strength exclusive to the oxygen atoms of the HSeO_3_^−^ anions (Figure 4b), which are better recipients than the halides. The [Cd(HSeO_3_)_2_*X*_2_]^2−^ layers in the en and pip structures are nearly the same (Figure 4c,d). As the quality of the (pipH_2_)[Cd(HSeO_3_)_2_Cl_2_] crystals was relatively low, several attempts were made to produce better ones. 

**Figure 5:**
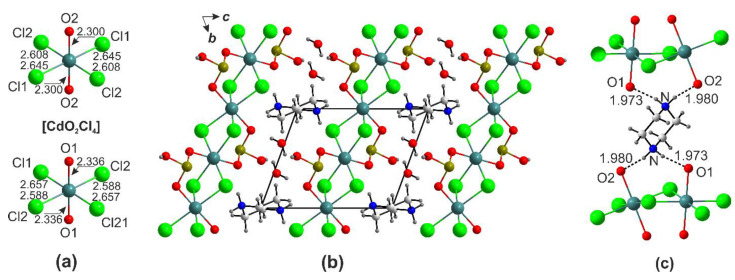
The crystal structure of (pipH_2_)[Cd(HSeO_3_)_2_Cl_2_]·2H_2_O. Cation coordination in the crystal structure of (pipH_2_)[Cd(HSeO_3_)_2_Cl_2_]·2H_2_O (**a**). Projection of the structure of (pipH_2_)[Cd(HSeO_3_)_2_Cl_2_]·2H_2_O onto the *bc* plane (**b**). Fragment of the structure with hydrogen bonds shown (**c**).

In a single case, a crystal with quite different metrics was picked, which was found to belong to a new compound (pipH_2_)[Cd(HSeO_3_)_2_Cl_2_]·2H_2_O (Table 3) with a related composition but a totally different structure (Figure 5).In this case, two symmetry-independent Cd^2+^ cations reside in *trans*-CdO_2_Cl_4_ octahedra (Figure 5a) which share common Cl···Cl edges to form chains (Figure 5b). The HSeO_3_^−^ anions do not form pairs; they act as hydrogen bond acceptors from the pipH_2_^2+^ cations (Figure 5c). The [CdCl_2_(HSeO_3_)_2_]^2−^ chains are also involved in hydrogen bonding with water molecules. 

### 2.3. Halide-Free Framework Structures

The organic templates also contribute to formation of halide-free complex selenite diselenites of the (diamineH_2_)[*M*(HSeO_3_)(Se_2_O_5_)]_2_ (*M* = Cd, Co, Mn, Zn) family (Table 3). These frameworks have been reported earlier for (NH_3_(CH_2_)_4_NH_3_)[*M*(HSeO_3_)(Se_2_O_5_)]_2_ (*M* = Zn, Co or Ni) series templated by tetramethylenediammonium cations [24]; these frameworks also seem to be rather elastic and readily incorporate cyclic piperazinium cations. 

The newcomer to this family is Mn^2+^. Overall, combinations of (protonated) SeO_3_^2−^ and Se_2_O_5_^2−^ are rather common in inorganic frameworks based on various *s*-, *d*-, and *f*-metals [25,26,27,28,29,30]. Their templating by various organic species is very likely to result in a variety of complex and elegant architectures. In the meantime, such species were not observed with the branched mpipH_2_^2+^ cation. A likely reason for this is the same as in the previous case: either the branched structure or the shape of the organic moiety. 

**Figure 6:**
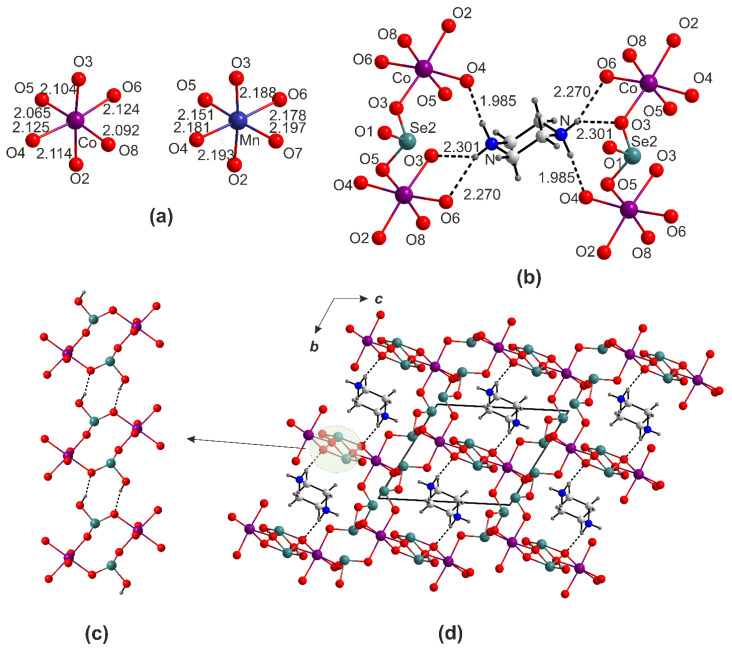
Cation coordination in the crystal structure of (pipH_2_)[M(HSeO_3_)(Se_2_O_5_)]_2_ (**a**). Hydrogen bonding systems (**b**,**c**). Projection of the structures of (pipH_2_)[M(HSeO_3_)(Se_2_O_5_)]_2_ onto the *bc* plane (**d**).

In the (pipH_2_)[*M*(HSeO_3_)(Se_2_O_5_)]_2_ (*M* = Co, Mn), the divalent cations center nearly regular *M*O_6_ octahedra (Figure 6a), with a mean distance of 2.1039(18) and 2.181(4) Å, respectively. Three symmetry-independent Se atoms contribute to the Se1Se2O_5_^2−^ and HSe3O_3_^−^ anions (Figure 6b). The latter associate into chains aligned along a via. The Se1O_3_ and Se2O_3_ polyhedra share vertices with the *M*O_6_ octahedra to form layers aligned in parallel (Figure 6c). Overall, a porous framework is formed (Figure 6d) with cavities filled by the piperazinium cations. The latter are for hydrogen bonds to the oxygen atoms of the framework. The channel size, estimated as the distance between the opposite oxygen atoms, is 4.13 × 5.58 Å. 

### 2.4. Ion-Molecular Crystals and Halometallates

**Figure 7:**
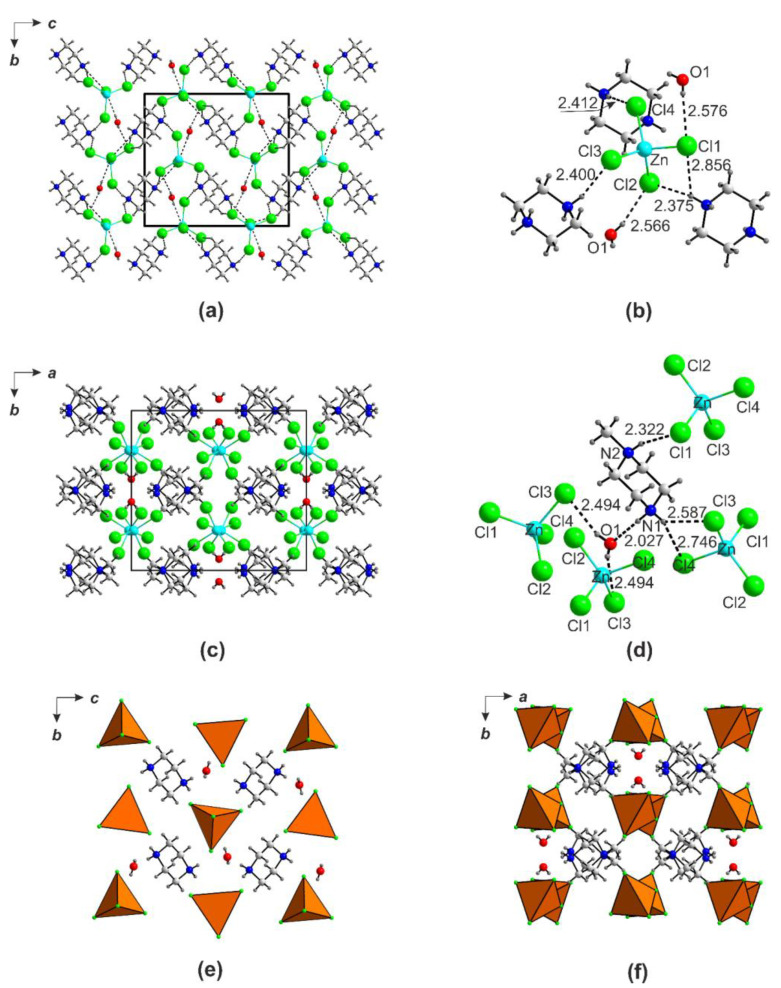
Projection of the structures of (pipH_2_)[ZnCl_4_](H_2_O) onto *bc* (**a**) and (mpipH_2_)ZnCl_4_·H_2_O onto *ab* (**c**) planes and hydrogen bonds system therein (**b**,**d**). Polyhedral representation of the crystal structure of (pipH_2_)[ZnCl_4_](H_2_O) onto the *cb* plane (**e**) and (mpipH_2_)ZnCl_4_·H_2_O (**f**) onto the *ab* plane (**f**).

Our previous studies have demonstrated that careful inspection of various crystals formed in selenite-containing systems may lead to discovery of unusual architectures, including first representatives of new intriguing families [31,32,33,34]. Therefore, all good quality crystals, including colorless (i.e., those evidently not containing Mn^2+^, Co^2+^, Ni^2+^, or Cu^2+^), were studied (Table 3). Formation of diammonium polyhalometallates was detected in many halide-containing runs, as illustrated by (pipH_2_)ZnCl_4_·H_2_O [35,36], (mpipH_2_)ZnCl_4_·H_2_O, non-centrosymmetric (pipH_2_)[ZnBr_4_], and (enH_2_)[Cd_2_Br_6_(H_2_O)], which contain both tetrahedrally and octahedrally coordinated cadmium [37]. The presence of excess acid (selenious of trifluoroacetic) liberates some free hydrohalic acid, which probably contributes to formation of these side products. In the structure of (pipH_2_)[ZnCl_4_](H_2_O) (Figure 7a), Zn^2+^ centers a [ZnCl_4_]^2−^ tetrahedron (<Zn-Cl> = 2.2763 Å) connected to pip by hydrogen bonds (Figure 7b). The Cl atoms accept two hydrogen bonds (2.856(4) and 2.375(2) Å) from the pipH_2_^2+^ cation, and one from the water molecule (2.566(3) Å). A close chemical composition was also found for the tetrachlorozincate of mpip (Figure 7c,d); however, due to the different size and shape of the cation, as well as the lower number of hydrogen bonds (three for mpipH_2_^2+^ vs. four for pipH_2_^2+^), the structure is also different (Figure 7e,f). The mpipH_2_^2+^ cation has three “active” nitrogen-bound hydrogens, which form two single hydrogen bonds and one bifurcated hydrogen bond accepted by Cl- from two different [ZnCl_4_]^2−^ anions and one water molecule. The hydrogen atoms of the latter also form bonds to the chlorozincate anions. 

**Figure 8:**
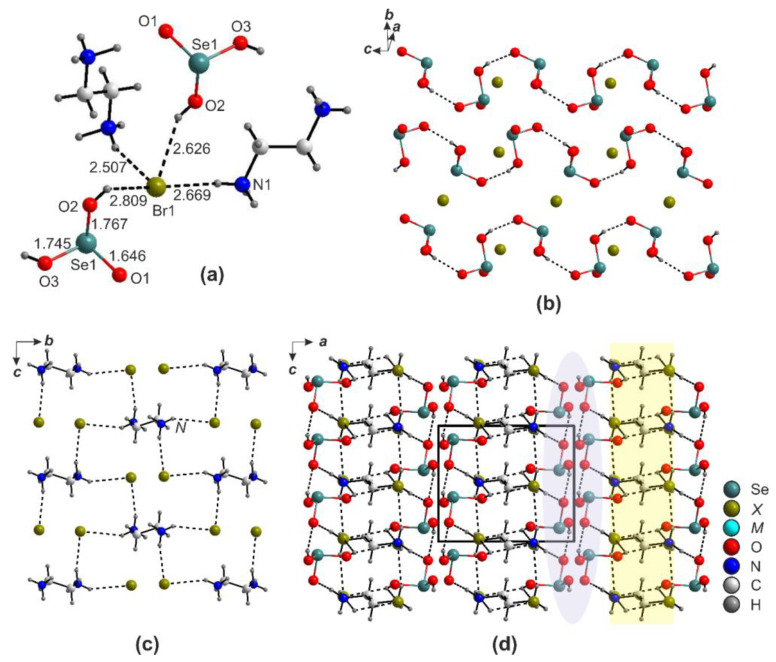
Hydrogen bonding in the structures of enH_2_*X*_2_·2H_2_SeO_3_ (*X* = Cl and Br) (**a**–**c**). Projection of the structure onto the *ac* plane (**d**).

The metal-free structure of (enH_2_)*X*_2_·2H_2_SeO_3_ (*X* = Cl and Br) (Figure 8) is the first “organic analog” of the *AX*·*n*H_2_SeO_3_ compounds (*A* is an alkali cation) [33,34], wherein the Se atom is also coordinated to three oxygen atoms, two of which are protonated to form the H_2_SeO_3_ molecule (Figure 8a). In the structure of halides (take *X* = Br for example), these species and the enH_2_^2+^ cations, as well as Br^−^, link via hydrogen bonding to form a framework wherein the organic and inorganic parts form sublayers distantly reminiscent of the *AX*·*n*H_2_SeO_3_ [33,34] (Figure 8a–c). The H_2_SeO_3_ molecules also form hydrogen bonds between each other (Figure 8d). 

**Figure 9:**
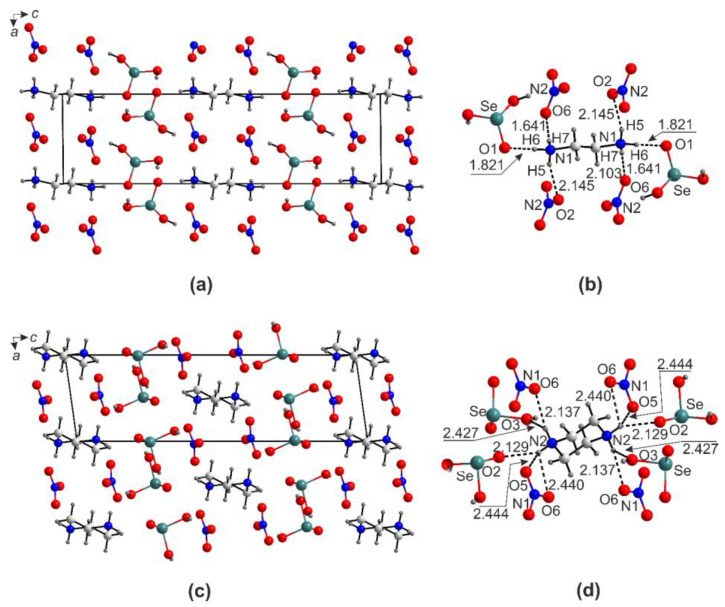
Projection of the structures of (enH_2_)(NO_3_)_2_·2H_2_SeO_3_ onto the *ac* (**a**) plane. Hydrogen bonding in the structures of (enH_2_)(NO_3_)_2_·2H_2_SeO_3_ (**b**). Projection of the structures of (pipH_2_)(NO_3_)_2_(H_2_SeO_3_)_2_ onto the *ac* (**c**) plane. Hydrogen bonding in the structures of (pipH_2_)(NO_3_)_2_(H_2_SeO_3_)_2_ (**d**).

The nitrate derivatives, (enH_2_)(NO_3_)_2_·2H_2_SeO_3_ and (pipH_2_)(NO_3_)_2_·2H_2_SeO_3_, have close chemical compositions but also different crystal structures (Figure 9). In the former structure (Figure 9a,c), the H_2_SeO_3_ species form chains aligned along *b*. They are decorated by the nitrate groups attached via hydrogen bonds to form ribbons. The enH_2_^2+^ cations are situated between these. As in the previous case, both architectures can be considered to be pseudo-layered, yet with a different topology; the layers are formed by changed and neutral moieties. Both organic species form hydrogen bonds to oxygens of both NO_3_^−^ and H_2_SeO_3_ moieties (Figure 9b,d). 

### 2.5. Structural Trends in Organic Hydrogen Selenites 

**Figure 10:**
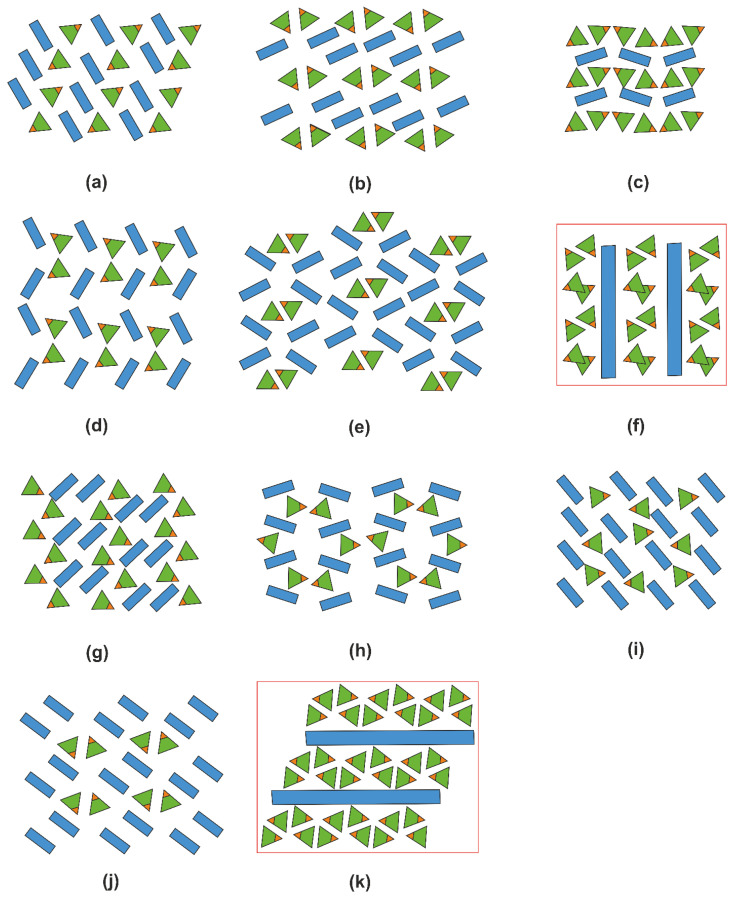
Diagrams for the organic hydrogen selenites (*B*H)^+^·HSeO_3_^−^. The structures reported here are framed in red. [C_2_H_6_NO_2_][C_2_H_5_NO_2_][HSeO_3_] [39] (**a**), [C_6_H_16_NO_3_][HSeO_3_] [40] (**b**), [C_6_H_16_N_2_][HSeO_3_] [41] (**c**), [C_4_H_6_N_3_O][HSeO_3_] [42] (**d**), [C_9_H_12_NO_2_][C_9_H_11_NO_2_][HSeO_3_] [43] (**e**), (enH_2_)(*M*(HSeO_3_)_2_*X*_2_) (**f**), [C_11_H_13_N_2_O_2_][HSeO_3_] [44] (**g**), [C_13_H_14_N_3_][HSeO_3_]H_2_O [45] (**h**), [C_8_H_20_N][HSeO_3_] [46] (**i**), [C_2_H_7_N_4_O][HSeO_3_] [47] (**j**), (pipH_2_)[Cd(HSeO_3_)_2_*X*_2_] (**k**). The organic species are identified in Table S1.

**Figure 11:**
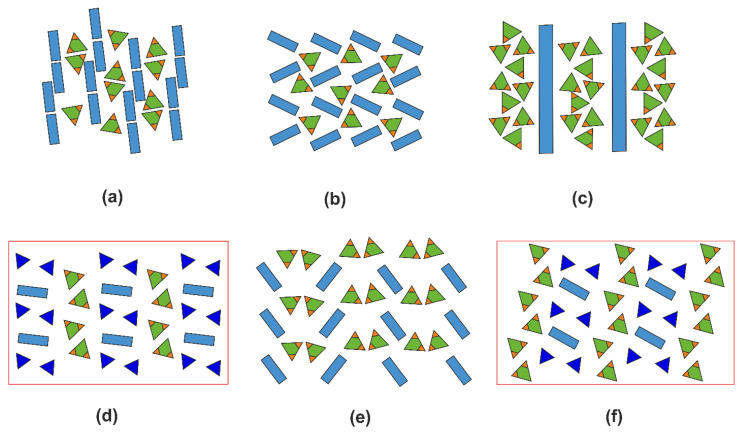
Diagrams for the structures containing organic matter and H_2_SeO_3_ molecules. As in the previous figure, the structures reported here are framed in red. [C_2_H_5_NO_2_][H_2_SeO_3_] [48] (**a**), [C_5_H_11_NO_2_][H_2_SeO_3_] [49] (**b**), [C_10_H_16_N][HSeO_3_][H_2_SeO_3_] [50] (**c**), (enH_2_)(NO_3_)_2_(H_2_SeO_3_)_2_ (**d**), (enH_2_)*X*_2_(H_2_SeO_3_)_2_ (**e**), (pipH_2_)(NO_3_)_2_(H_2_SeO_3_)_2_ (**f**). The organic species are identified in Table S1.

**Figure 12:**
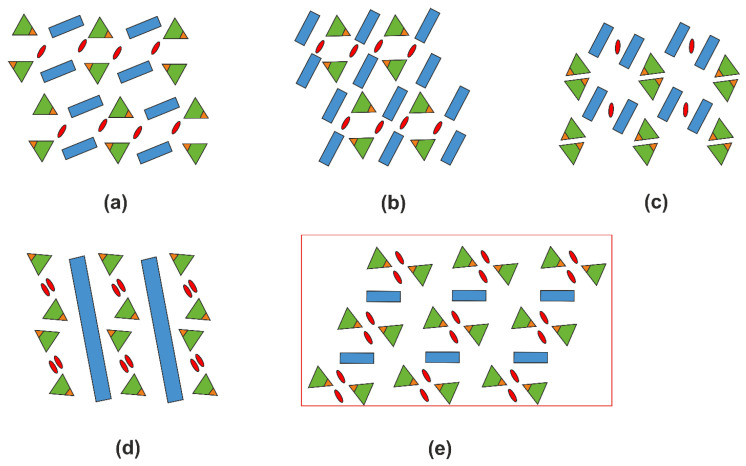
Diagrams for the structures containing extra water molecules (as in the previous figure, the structures reported here are framed in red): [C_7_H_8_NO_2_][HSeO_3_]H_2_O [51] (**a**), [C_6_H_8_N][HSeO_3_][H_2_O] [52] (**b**), [C_6_H_15_N_4_O_2_][HSeO_3_]·0.15H_2_O [53] (**c**), and [C_36_H_30_NP_2_][HSeO_3_][CH_2_Cl_2_][H_2_O] [54] (**d**), (pipH_2_)[Cd(HSeO_3_)_2_Cl_2_]·2H_2_O (**e**). The organic species are identified in Table S1.

Table S1 contains 24 entries for hydrogen selenites containing organic cations. To determine the topology of mutual positioning of the organic and inorganic parts, we used the approach developed in [38]. For each structure, a diagram is constructed where green triangles designate the partially or fully protonated SeO_3_ groups, with the OH vertices highlighted in brown, the organic species are shown as blue rectangles, and the water molecules are shown as red ovals. These structures can be divided into three groups: those containing only HSeO_3_^−^ (Figure 10), those also containing H_2_SeO_3_ molecules (Figure 11), and those containing crystal water molecules (Figure 12). 

In the structures of organic hydrogen selenites (*B*H)^+^·HSeO_3_^−^ (where *B* designates protonated organic matter), one can clearly distinguish the segregation of organic and inorganic species into layer-like areas, irrespective of the size and shape of the former. In the latter, the HSeO_3_^−^ species aggregate into dimers via hydrogen bonding. This trend is visible even in such complex structures as that of the phenylalanine and guanylurea derivatives, [C_9_H_12_NO_2_][C_9_H_11_NO_2_][HSeO_3_] and [C_2_H_7_N_4_O][HSeO_3_], where the hydroselenite groups can be alternatively considered as being embedded into the organic sublattice. 

The trend in segregation of the organic and inorganic matter can also be followed in the few structures containing neutral molecules of selenious acid (Figure 11). When both constituents are neutral (e.g., in co-crystals of amino acids), we can consider the result to be a hybrid organo-inorganic composite. A more complex pattern is observed for (enH_2_)(NO_3_)_2_·2H_2_SeO_3_ and (pipH_2_)(NO_3_)_2_·2H_2_SeO_3_, where there are several hydrogen bond donors and acceptors. 

Addition of water molecules to these organo-inorganic architectures results in “delamination” into three types of layers; those formed by water molecules are either sandwiched between the organic matter layers or between those and the selenite groups (Figure 12). Note that the number of these entries is very small; no structure is known containing neutral molecules of both selenious acid and water. 

Overall, the structures discussed here can be considered as complex organo-inorganic architectures. The segregation of the organic and inorganic parts can be understood considering that the former species aggregate due to the dominating Van der Waals (hydrophobic) interactions; they interact with the latter only via hydrogen bonding. The donors of “linking” hydrogen bonds are the protonated nitrogen atoms of the organic matter, while the recipients are the inorganic anions, including HSeO_3_^−^ and H_2_SeO_3_; the latter also interact via hydrogen bonding, but only between each other (Figure 3e), which is likely the cause of their aggregation. The size and shape of the organic molecules do not affect the type of hydrogen bonding. 

### 2.6. Overall Remarks

The new compounds observed in our studies form several new families containing both known and new structure types, and their diversity and elegance is quite amazing. It is also evident that at least one half of these can be considered to be the first representatives of significantly more numerous groups. Due to almost unlimited diversity of organic hydrogen bond donors with both rigid and flexible backbones and, correspondingly, the topology of hydrogen bond directions, the structural chemistry of their derivatives also seems to be hardly restricted. However, some relationships can be traced and some new families predicted. 

The most common structure, previously reported for (enH_2_)[*M*(HSeO_3_)_2_Cl_2_] (*M* = Cd [21], Cu, and Co [19]) is observed for the majority of metal di-cations studied, including bromides and compounds of zinc. Three newcomers to this family are Mn^2+^, Mg^2+^, and Ni^2+^, although the latter two only with some reluctance. The electronic structure of the metal di-cation is therefore not sufficient; the size of Mg^2+^ is relatively close to Zn^2+^ or Mn^2+^ [55]. We may tentatively assume, however, that because its electronic structure is different, the lack of *d*-orbitals hinders formation of Mg–X bonds even in the presence of a large excess of competing water molecules. Examples are quite abundant when, even in halide-rich media, products of ambient-temperature crystal hydrates mostly contain the initial [Mg(H_2_O)_6_]^2+^ cations. Illustrative examples are provided by the structures of the [Mg(H_2_O)_6_][ZnCl_4_] and [Mg(H_2_O)_6_]_2_[MnCl_6_] compounds [56,57], wherein Mg^2+^ and Zn^2+^ or Mn^2+^ are bonded selectively to the water molecules and chloride anions, respectively, as well as by synthetic analogs of carnallite [58]. The [Ni(H_2_O)_6_]^2+^ cation present in the initial dilute solution does not undergo a Jahn–Teller distortion (high-spin *d*^8^ configuration, *t*_2*g*_^6^*e_g_*^2^) and is expected to be more inert (at least, under ambient conditions) towards ligand exchange compared to Mn^2+^ and Zn^2+^, with CFSE = 0 and Co^2+^ and Cu^2+^ exhibiting an essential Jahn–Teller distortion. An illustration can be provided by the structures of NiCd_2_Cl_6_·12H_2_O [59], which also contains isolated [Ni(H_2_O)_6_]^2+^ octahedra in the cavities of a complex Cd^2+^–Cl^−^–H_2_O framework, wherein Cd^2+^ easily adopts a mixed-ligand coordination. This may explain the absence of the corresponding (enH_2_)[M(HSeO_3_)_2_Br_2_] species with M = Mg and Ni. 

The non-rigid and relatively small ethylenediammonium cation, which forms as many as six hydrogen bonds, easily fits into the space between the hydroselenite-halide [*M*(HSeO_3_)_2_*X*_2_]^2−^ layers. With more voluminous and more rigid piperazinium, the layered structure is observed only for Cu^2+^ (which is the commonest contributor to the layered hydroselenite family, vide supra) and the largest Cd^2+^. Even then, the layered hydroselenite structure seems to lie on the stability edge, as exemplified by the formation of the structurally unrelated dihydrate. With smaller Mn^2+^, Co^2+^, or Zn^2+^, only the “microporous” hydroselenite-diselenites are formed; Mg^2+^ also does not contribute to this family. This 3D framework is also relatively flexible and can host both linear-chain (CH_2_)_4_(NH_3_)_2_^2+^ and piperazinium cations. Finally, the branched N-methylpiperazinium cation probably has no chance to contribute to any of these, and simpler and unconstrained structures of tetrahalometallates are formed instead; most starting solutions containing this cation produced no crystals at all. In targeted attempts to produce crystals of (*B*H_2_)(NO_3_)_2_·2H_2_SeO_3_ with *B* = pip and mpip, the formed solution produced the target crystals within several weeks, while the latter remained viscous. It should be noted, however, that nearly all solutions containing selenious acid become syrupy, and nearly glassy at the last stages of evaporation, which probably hinders crystallization. 

The reluctance of most *M*^2+^ cations to coordinate NO_3_^−^ in aqueous solutions can be expected given the high hygroscopicity of the respective nitrates (those of Mg and Mn deliquesce quite rapidly when exposed to air). It is also possible that cationic species with larger effective radii should be present, suggesting the ammonium “head” of the diamine comparable in size to NH_4_^+^ and K^+^. We note that the former contributes to a relatively complex, strongly hydrogen-bonded [(NH_4_)_3_(NO_3_)][Cu(HSeO_3_)_2_(NO_3_)_2_] layered architecture [9], while the latter does not contribute at all [14]. It is possible that ammonium nitrate-related architectures can be produced using polyethylenepolyammonium species instead of discrete ammonium cations, e.g., [(dienH_3_)(NO_3_)][Cu(HSeO_3_)_2_(NO_3_)_2_] or [(trienH_4_)(NO_3_)_2_][Cu(HSeO_3_)_2_(NO_3_)_2_] (dien = diethylenetriamine, trien = triethylenetetramine), etc. 

The structures of the organic species containing both selenium and organics can thus be divided into three groups: (i) the “layered hydroselenites”, containing all constituents, (ii) the halide-free “microporous structures”, and (iii) the metal-free “ion-molecular crystals”. For the first family, we predict the existence of new members with relatively flexible non-branched or short-branched backbones containing ammonium groups at the opposite ends which would fit the size of the [*M*(HSeO_3_)_2_] grids, e.g., α,ω-polymethylenediammonium cations, ^+^H_3_N(CH_2_)*_n_*NH_3_^+^. Note, however, that at least some of these can also contribute to the halide-free structures [26]. Formation of double Langmuir-like layers of alkylammonium cations, similar to Cs_2_[Co(HSeO_3_)_2_Cl_2_] [17], is also possible. As the Cu- and Co-based compounds exhibit magnetic ordering [19], it may be of interest to see how the intralayer interactions would be affected by varying the interlayer distances by changing the spacer size. Some of these investigations are now in progress. 

The second family of structures contains the [*M*_2_(HSeO_3_)_2_(Se_2_O_5_)_2_]^2−^ frameworks with a limited inner space, so more voluminous species such as mpipH_2_^2+^ are probably too large to fit therein; the alternative found in our experiments is the formation of simpler structures of halometallates. Therefore, long chains of larger-cycle diamines (or polyamines) will most likely not be “admitted”, including for compounds with branched backbones. Other topologies are known, however, e.g., based on vanadyl cations, where such species act as templates for chiral porous frameworks [60]. 

The third group, represented as yet by a pair of isostructural compounds, enH_2_*X*_2_·2H_2_SeO_3_ (*X* = Cl and Br) and the non-analogous nitrate enH_2_(NO_3_)_2_·2H_2_SeO_3_, also promises to be diverse as the hydrogen-bonded nets of H_2_SeO_3_ molecules and are expected to be the very flexible and adaptable. Another example is (pipH_2_)(NO_3_)_2_·2H_2_SeO_3_. Due to the unlimited variability of the organic species, which need not necessarily be hydrogen bond donors or acceptors (similar to alkali cations), one can expect formation of various structures, including non-centrosymmetric structures, by careful choice of the corresponding templates. Formation of non-centrosymmetric organically templated inorganic selenites has been documented [8,60]; further perspectives in this direction are quite promising. Note that while nitrates and halides can contribute to similar structures when these anions form purely ionic bonds to the cationic species (alkali cations), the analogies disappear when hydrogen bonding is enforced. The very symmetrical halide anions can form hydrogen bonds in all possible directions, while the rigid planar nitrate group has a fixed geometry of hydrogen bond acceptors. 

The few structures of selenium-free halometallates observed in our work also fit very well into the structural trends observed in this relatively well-addressed family. The non-centrosymmetric structure of (pipH_2_)[ZnBr_4_] is a full analog of (pipH_2_)[CoBr_4_] [61] and (pipH_2_)[CdI_4_] [35]. The (pipH_2_)[ZnCl_4_](H_2_O) is in turn isostructural to (pipH_2_)[CdBr_4_](H_2_O) [62]; therefore, the structures and hydration state of compounds containing [ZnCl_4_]^2−^ and [ZnBr_4_]^2−^ (or [CoBr_4_]^2−^) are similar to those containing [CdBr_4_]^2−^ and [CdI_4_]^2−^ or [HgI_4_]^2−^, respectively, which points at the *X*^−^/*M*^2+^ size ratio as the structure-driving factor. Hence, the (mpipH_2_)[ZnCl_4_](H_2_O) is expected to be isostructural to the (mpipH_2_)[CdBr_4_](H_2_O) reported in [63]; however, the latter was not structurally characterized. Thus, the family hosts at least four non-centrosymmetric and one complex layer structure. Its future also seems intriguing. 

## 3. Materials and Methods

### 3.1. Synthesis

The starting compounds were the organic diamines, selenious acid, and corresponding metal halides or nitrates. Unimolar working solutions of all compounds were prepared first. Next, *MX*_2_, H_2_SeO_3_, and diamine solutions were mixed together to provide a 1:1:3 molar ratio. Generally, 1 mL of *MX*_2_ solution was mixed with 3 mL of 1*M* H_2_SeO_3_. Next, 1 mL of the diamine solutions was added dropwise upon stirring with a small glass bar. The 50% excess of the selenious acid was shown in [10,13,21] to be necessary to suppress formation of insoluble metal selenites and competitive formation of halometallates, which easily form if extra hydrohalic acid is added [13]. Commonly, precipitation or turbidity due to formation of *M*SeO_3_·aq was observed after diamine was introduced, which mostly disappeared after stirring and heating to 50–60 °C for 5 to 10 min on a hotplate. Until clearance, several drops of water were added to keep the solution volume constant. With *M* = Cu or Cd, which form less soluble selenites, the flaky green and white (respectively) precipitates were dissolved after heating and adding 0.1 to 0.7 mL of trifluoroacetic acid in the case of halides and 0.1–0.5 mL of 50% nitric acid for nitrates, according to the protocols developed earlier [13,15]. (In one or two cases, traces of CdSeO_3_·aq persisted but dissolved over time when crystals of the targeted compounds started to form.) The halide solutions (colorless in case of *M*^II^ = Mg, Zn and Cd, initially pink for Mn, violet for Co, green in the case of Ni, and blue in the case of Cu) were left to evaporate at ambient conditions. Crystallization started in a few weeks, after the color of halide-bearing solutions containing Co and Cu turned lilac (Co), green (Cu–Cl), or brownish (Cu–Br); the color of the Cu- and Co-containing crystals was bluish-green and dark violet, respectively. The intermediate change in solution colors likely indicates transient formation of [*MX*_4_^2−^] due to free hydrohalic acid present in solution. No color change was observed for the solutions containing Mn and Ni (as well as in all experiments with nitrates). The Mn- and Ni-bearing crystals were grayish-pink and brownish yellow. The crystals grown in Mg-containing solutions were of relatively low quality. Sometimes, small amounts of red selenium were precipitated due to reduction of selenious acid, which did not affect the formation and growth of the target crystals. The exception was dmeda, which was rapidly oxidized by selenious acid; this compound was therefore excluded from further studies. The crystals were collected and kept in closed vials under a drop of the mother liquor. Further evaporation of the mother liquors led to repeated deposition of crystals with similar color and appearance. The majority of these runs provided the target hydroselenite halides for en, just a few for pip, and none for mpip. In most cases, crystals of some side products were also observed, which were formed either due to the off-stoichiometric reagents ratio of the initial charge, or instead of the targeted compound. Based on the combination of diamine, metal cation, and anion, these side products may contain either all constituents, or only some of these; those exhibiting new compositions and/or structures are discussed in detail.

A related pattern was observed for the nitrate solutions, except that the color of the crystals corresponded to those of the hydrated *M*^2+^ cations. The only exception was observed in the case of Mn^2+^, which produced black needles of low quality; such a color change is indicative of oxidation of Mn^2+^ into Mn^3+^ or Mn^4+^. This compound will be addressed elsewhere. In these cases, no target compound was observed; crystals of a new compound (enH_2_)(NO_3_)_2_∙2H_2_SeO_3_ were produced with all runs with en. With pip and mpip, no crystalline produces were observed for the nitrate runs.

As in the previous studies, in the last stages the liquors commonly became dark and viscous, produced no more crystals, and were discarded. It is worth noting that crystals were the first to form with en, next with pip, and with greater difficulty in the case of mpip; some solutions containing mpip produced no crystals but only viscous residues.

According to the classification suggested above, the produced species were: (i) (enH_2_)[*M*(HSeO_3_)_2_*X*_2_] (*M* = Cd, Co, Cu, Mn, Zn, Ni; *X* = Cl, Br) and (pipH_2_)[*M*(HSeO_3_)_2_*X*_2_] (M = Cd, *X* = Cl and Br; *M* = Cu, *X* = Cl; (ii) (pipH_2_)[*M*(HSeO_3_)(Se_2_O_5_)]_2_ (M = Mn, Co, Zn); (iii) enH_2_*X*_2_·2H_2_SeO_3_ (*X* = Cl, Br, and NO_3_) and (pipH_2_)(NO_3_)_2_·2H_2_SeO_3_; and (iv) (pipH_2_)[ZnCl_4_](H_2_O), (mpipH_2_)[ZnCl_4_](H_2_O), and (pipH_2_)[ZnBr_4_]. The side products most likely come from the off-stoichiometric ratio of the initial solutions; these were observed in most deposited samples. The three new compounds enH_2_*X*_2_∙2H_2_SeO_3_ (*X* = Cl, Br, and NO_3_) were prepared in targeted runs by mixing the 1*M* working solutions of en, H*X*, and H_2_SeO_3_ taken in a 1:2.1:2 volume ratio; large (up to several mm) colorless crystals were produced after several weeks of evaporation at ambient conditions.

### 3.2. Single Crystal X-ray Study

The collected crystals were taken out of the mother liquors, attached to glass fibers, and transferred to a Rigaku XtaLAB Synergy-S diffractometer (Tokyo, Japan) equipped with a PhotonJet-S detector (Tokyo, Japan) operating with MoKα radiation at 50 kV and 1 mA. A single crystal was chosen and more than a hemisphere of data was collected with a frame width of 0.5° in ω, and 5–15 s was spent counting for each frame. The data were integrated and corrected for absorption applying a multiscan type model using the Rigaku Oxford Diffraction programs CrysAlis Pro (Rigaku OD, 2015) (Tokyo, Japan). The experiments were performed at 150 K. The unit cell parameters were calculated by the least-squares method. The structures were solved by direct methods using WinGX version 2020.1 (Glasgow, UK) [64] and Olex2 version 1.3.0 (Regensburg, Germany) [65] software. The final solutions include the coordinates and anisotropic thermal parameters of atoms except for hydrogens. Hydrogen atoms were located using the mathematical part of the Olex2 program. 

Experimental data for the (enH_2_)[*M*(HSeO_3_)_2_*X*_2_] series are collected in Table 1 (except *M* = Mg, where the crystals were of low quality). All these compounds are isostructural; some trends are discussed below. The corresponding data for the piperazinium compounds are listed in Table 3. In contrast to the alkali-bearing family, in no case did we observe formation of organic-containing layered hydroselenite nitrates; the organic and inorganic matter crystallized separately yielded *M*SeO_3_⋅aq (or *M*(HSeO_3_)_2_); and with en and pip, new compounds (*B*H_2_)(NO_3_)_2_·2H_2_SeO_3_ were observed, which are considered below. The simplest side results are also discussed here, while some others with 3*D* structures will be reported in separate contributions.

## Figures and Tables

**Table 1:** Crystallographic and refinement parameters for (enH_2_)[*M*(HSeO_3_)_2_*X*_2_]: (enH_2_)[Cd(HSeO_3_)_2_Br_2_] (**1**), (enH_2_)[Cd(HSeO_3_)_2_Cl_2_] (**2**), (enH_2_)[Co(HSeO_3_)_2_Br_2_] (**3**), (enH_2_)[Co(HSeO_3_)_2_Cl_2_] (**4**), (enH_2_)[Cu(HSeO_3_)_2_Br_2_] (**5**), (enH_2_)[Cu(HSeO_3_)_2_Cl_2_] (**6**), (enH_2_)[Mn(HSeO_3_)_2_Br_2_] (**7**), (enH_2_)[Mn(HSeO_3_)_2_Cl_2_] (**8**), (enH_2_)[Zn(HSeO_3_)_2_Br_2_] (**9**), (enH_2_)[Zn(HSeO_3_)_2_Cl_2_] (**10**), (enH_2_)[Ni(HSeO_3_)_2_Cl_2_] (**11**).

	1	2	3	4	5	6	7	8	9	10	11
Radiation		MoKα, 0.71073									
Crystal system		monoclinic									
Space group		*P*2_1_/*c*									
*a* (Å)	8.8222(4)	8.2957(5)	8.9395(6)	8.6750(4)	9.1833(4)	8.9953(3)	8.9465(4)	8.2958(3)	9.1862(4)	8.6699(4)	8.6420(8)
*b* (Å)	7.6140(3)	7.8094(3)	7.3147(4)	7.3277(3)	7.1548(3)	7.1677(2)	7.4460(3)	7.6712(2)	7.1516(2)	7.3448(2)	7.3081(4)
*c* (Å)	10.2481(5)	9.9433(5)	9.7558(8)	9.6812(4)	9.4845(5)	9.3819(4)	10.0240(5)	9.5977(4)	9.4849(4)	9.6996(4)	9.6000(9)
β (°)	114.196(6)	112.430(6)	111.904(9)	113.093(6)	110.608(5)	111.215(4)	112.720(6)	111.293(4)	110.624(4)	113.228(5)	112.999(11)
Volume (Å^3^)	627.91(6)	595.44(6)	591.88(8)	566.10(4)	583.30(5)	563.91(3)	615.94(5)	569.09(3)	583.19(4)	567.59(4)	558.11(8)
*Dcalc* (g/cm^3^)	3.122	2.726	3.012	2.628	3.083	2.665	2.829	2.590	3.094	2.658	2.664
*θ* range (°)	3.45–33.64	3.41–33.26	3.58–38.06	3.60–35.53	3.66–33.49	3.68–35.65	3.51–33.72	3.50–35.49	3.66–35.66	3.59–35.42	3.68–27.99
*h*, *k*, *l* ranges	12→−12 11→−9 15→−14	12→−12 8→−12 14→−13	15→−15 11→−12 15→−16	−9→13 −9→11 −15→14	−13→12 −10→10 −12→12	−14→13 −11→11 −15→15	−13→12 10→−8 14→−13	−13→11 −10→12 −13→15	−14→14 −11→11 −14→15	−11→14 −11→7 −15→14	11→−11 9→−9 12→−12
Total reflections collected	2119	2019	4713	2319	1992	2395	2091	2362	2414	2339	1349
Unique reflections (*Rint*)	1885 (0.032)	1702 (0.031)	3061 (0.08)	2009 (0.043)	1710 (0.054)	2101 (0.059)	1791 (0.035)	2109 (0.031)	2128 (0.042)	2089 (0.041)	1048 (0.075)
*R*1[*F* > 4*σF*], *wR*1[*F* > 4*σF*]	0.023 (0.051)	0.025 (0.053)	0.047 (0.084)	0.029 (0.056)	0.035 (0.070)	0.024 (0.057)	0.030 (0.073)	0.022 (0.049)	0.028 (0.062)	0.028 (0.058)	0.039 (0.076)
Rall, wRall	0.028 (0.052)	0.033 (0.054)	0.082 (0.092)	0.036 (0.058)	0.043 (0.073)	0.030 (0.058)	0.037 (0.075)	0.026 (0.050)	0.035 (0.063)	0.033 (0.059)	0.058 (0.081)
Goodness of fit	1.053	0.984	0.989	1.056	1.095	1.026	1.107	0.981	1.125	1.047	1.058
CCDC number	2,271,293	2,271,295	2,271,296	2,271,297	2,271,298	2,271,307	2,271,300	2,271,301	2,271,306	2,271,305	2,271,304

**Table 2:** Geometrical parameters of *M*O_4_*X*_2_ polyhedra in (enH_2_)[*M*(HSeO_3_)_2_*X*_2_].

Polyhedron	CdO_4_Br_2_	CdO_4_Cl_2_	CoO_4_Cl_2_	CuO_4_Br_2_	CuO_4_Cl_2_	MnO_4_Br_2_	MnO_4_Cl_2_	ZnO_4_Br_2_	ZnO_4_Cl_2_	NiO_4_Cl_2_
Bond valence for *M*^2+^	2.04	2.08	2.00	1.89	2.09	2.11	2.04	2.09	1.90	2.01
Average bond length, Å	2.4387	2.3950	2.2243	2.2807	2.2329	2.3587	2.3032	2.2806	2.2318	2.1973
Polyhedral volume, Å^3^	19.063	18.177	14.453	15.083	14.271	17.124	16.131	15.081	14.611	13.9603
Distortion index (bond length)	0.0768	0.0546	0.0884	0.1686	0.1532	0.1086	0.0735	0.1689	0.0866	0.08296

**Table 3:** Crystallographic and refinement parameters for (pipH_2_)[Cd(HSeO_3_)_2_Cl_2_] (**12**), (pipH_2_)[Cd(HSeO_3_)_2_Br_2_] (**13**), (pipH_2_)[Co(HSeO_3_)(Se_2_O_5_)]_2_ (**14**), (pipH_2_)[Mn(HSeO_3_)(Se_2_O_5_)]_2_ (**15**), (enH_2_)(H_2_SeO_3_)Br_2_ (**16**), (pipH_2_)(ZnCl_4_)(H_2_O) (**17**), (mpipH_2_)ZnCl_4_(H_2_O) (**18**), (enH_2_)(NO_3_)_2_ 2H_2_SeO_3_ (**19**), (pipH_2_)[Cd(HSeO_3_)_2_Cl_2_](H_2_O)_2_ (**20**), (pipH_2_)(NO_3_)_2_·2H_2_SeO_3_ (**21**).

	12	13	14	15	16	17	18	19	20	21
Radiation	MoK_α_, 0.71073									
Crystal system	triclinic				monoclinic				triclinic	monoclinic
Space group	*P*-1				*P*2_1_/c			*P*2_1_/c	*P*-1	*P*2_1_/*n*
*a* (Å)/α (°)	7.6207(3)/ 77.077(4)	7.7373(2)/ 76.646(3)	7.4411(3)/ 114.766(4)	7.5632(3)/ 115.300(4)	8.0012(5)	6.5391(2)	14.2117(3)	5.9386(3)	7.14040(10)/ 107.419(2)	5.61000(10)
*b* (Å)/β (°)	9.4337(4)/ 88.138(4)	9.4999(3)/ 88.778(2)	8.5623(4)/ 93.329(3)	8.7844(3)/ 92.985(3)	11.1514(7)/ 90.012(5)	12.6400(3)/ 92.567(2)	12.6651(3)/ 102.492(2)	5.2221(3)/ 90.669(4)	7.5642(2)/ 95.832(2)	6.8184(2)/ 98.024(2)
*c* (Å)/γ (°)	10.0207(4)/ 68.552(4)	10.1340(3)/ 68.472(3)	9.2460(4)/ 114.568(4)	9.4451(3)/ 114.829(4)	6.8168(4)	13.8009(4)	13.6203(3)	21.0732(11)	9.0469(2)/ 106.206(2)	18.7159(5)
Volume (Å^3^)/Z	652.57(5)	672.53(4)	466.85(4)	493.96(4)	608.23(6)	1139.56(6)	2393.52(9)	653.48(6)	438.464(18)	708.90(3)
*Dcalc* (g/cm^3^)		3.043	3.331	3.068	2.620	3.053	2.906	2.257	2.828	2.202
*θ* range (°)		3.38–27.50	3.43–33.64	3.38–33.49	3.50–35.66	3.37–35.65	3.49–35.44	3.43–33.60	3.47–38.02	3.68–38.09
*h*, *k*, *l* ranges		10→−10 12→−12 13→−13	11→−10 12→−13 13→−13	10→−11 13→−13 14→−13	12→−10 15→−17 6→−10	10→−10 13→−20 20→−22	21→−22 20→−18 22→−20	7→−8 7→−7 27→−32	12→−12 12→−12 15→−14	9→−8 11→−10 31→−27
Total reflections collected		3084	3120	3331	2335	4741	4979	2234	4484	3622
Unique reflections (*Rint*)		2748 (0.057)	2624 (0.031)	2724 (0.042)	1887 (0.036)	4069 (0.035)	4379 (0.025)	1827 (0.06)	3997 (0.034)	3046 (0.037)
*R*1[*F* > 4*σF*], *wR*1[*F* > 4*σF*]		0.035 (0.085)	0.025 (0.045)	0.038 (0.087)	0.033 (0.076)	0.025 (0.049)	0.019 (0.042)	0.053 (0.14)	0.025 (0.56)	0.028 (0.62)
Rall, wRall		0.040 (0.087)	0.033 (0.046)	0.049 (0.089)	0.045 (0.079)	0.033 (0.051)	0.024 (0.043)	0.064 (0.15)	0.030 (0.57)	0.038 (0.65)
Goodness-of-fit		1.024	1.030	1.150	1.004	1.055	1.056	1.081	1.026	1.029
CCDC number	*	2,275,315	2,275,344	2,275,377	2,275,047	2,275,306	2,275,269	2,275,256	2,275,336	2,275,366

* only unit-cell parameters are given.

## Data Availability

Data can be obtained from the authors upon request.

## Supplementary Materials

The supporting information can be downloaded at: https://www.mdpi.com/article/10.3390/ijms241814202/s1. References [66,67,68] are cited in the Supplementary Material.
