# Supplementary material for: Protonated Organic Diamines as Templates for Layered and Microporous Structures: Synthesis, Crystal Chemistry, and Structural Trends among the Compounds Formed in Aqueous Systems Transition Metal Halide or Nitrate–Diamine–Selenious Acid
Source: Int J Mol Sci. 2023 Sep 17;24(18):14202. doi: 10.3390/ijms241814202 (PMC10532228; doi:10.3390/ijms241814202)
Supplement: Supplementary file 1 [file ijms-24-14202-s001.zip › ijms-2500088-supplementary.pdf]

Table S1. Crystallographic data for known hydrogen selenites with organic molecules.

Chemical formula	Space group	*a*, Å	*α*,°	*b*, Å	*β*, °	*c*, Å	*γ*, °	Reference
$[C_6H_{15}N_4O_2][HSeO_3]$ 0.15(H_2O)	*C*2	22.493(5)		5.1624(13)	95.68(3)	9.730(4)		Matos Gomes et al., 2001 [17]
$[C_9H_{12}NO_2][C_9H_{11}NO_2][HSeO_3]$	*P*1	11.0507(4)	107.867(2)	12.8611(5)	110.392(2)	15.8820(6)	90.017(2)	Gharzaryan et al., 2010 [3]
$[C_8H_{20}N][HSeO_3]$	*P*-1	7.926(6)	80.64(1)	8.909(7)	65.15(1)	9.146(7)	77.19(2)	Wang et al., 2006 [18]
$[C_2H_7N_4O][HSeO_3]$	*P*-1	6.7643(4)	63.021(1)	7.9045(5)	81.414(1)	8.2612(5)	72.200(1)	Ritchie et al., 2003 [13]
$[CH_6N_3][HSeO_3]$	*P*2$_1$	4.145(2)		7.851(3)	98.77(3)	9.154(4)		Krumbe et al., 1987 [5]
$[HSeO_3][C_2H_6NO_2][C_2H_5NO_2]$	*P*2$_1$/*c*	12.2651(7)		4.8079(6)	122.745(4)	19.955(1)		Nemec et al., 1998 [7]
$[C_{11}H_{13}N_2O_2][HSeO_3]$	*P*2$_1$2$_1$2$_1$	5.4406(6)		8.2436(6)		27.539(3)		Paixao et al., 2006 [11]
$[C_7H_8NO_2][HSeO_3][H_2O]$	*P*-1	4.6718(6)	104.395(12)	9.3967(15)	92.705(11)	12.7992(14)	103.109(13)	Kamali et al., 2018 [4]
$[C_{13}H_{14}N_3][HSeO_3][H_2O]$	*P*2$_1$/*n*	6.360(1)		19.272(2)	93.75(1)	12.604(2)		Paixao et al., 1997 [12]
$[C_6H_8N][HSeO_3][H_2O]$	*P*-1	4.7212(2)	107.297(3)	9.3681(3)	92.294(3)	11.1290(3)	103.549(3)	Takouachet et al., 2016 [14]
$[C_6H_8N][HSeO_3][H_2O]$	*P*1	4.7423(3)	107.271(6)	9.4263(8)	92.652(6)	11.1599(7)	103.370(7)	Takouachet et al., 2016 [14]
$[C_{20}H_{15}N_4][HSeO_3]$ 2(H_2O)	*P*2$_1$/*c*	18.708(2)		14.320(1)	92.89(1)	7.349(3)		Chen et al., 2010 [2]
$[C_4H_6N_3O][HSeO_3]$	*Pca*2$_1$	7.0051(3)		8.6342(2)		12.7131(3)		Takouachet et al., 2014 [15]
$[C_6H_{16}N_2][HSeO_3]$	*Pbcn*	14.682(2)		6.491(1)		13.065(2)		Nemec et al., 2001 [8]
$[C_6H_{16}NO_3][HSeO_3]$	*P*2$_1$	8.043(1)		5.3468(9)	93.66(1)	12.543(2)		Lukevics et al., 2002 [6]
$[C_{36}H_{30}NP_2][HSeO_3][CH_2Cl_2][H_2O]$	*P*2$_1$/*c*	10.8664(11)		12.6743(13)	95.727(2)	26.029(3)		Canossa et al., 2018 [1]
$[C_{10}H_{16}N][HSeO_3][H_2SeO_3]$	*Pbca*	9.840(1)		24.416(5)		12.2489(8)		Matos Gomes et al., 1995 [16]
$[C_5H_{11}NO_2][HSeO_3]$	*Fdd*2	28.042(2)		21.815(2)		6.073(2)		Paixao et al., 1997 [11]
$[C_2H_5NO_2][HSeO_3]$	*P*2$_1$/*n*	5.049(1)		13.677(2)	94.92(1)	8.664(1)		Ondracek et al., 1992 [9]
$[C_{10}H_{16}N][HSeO_3][H_2SeO_3]$	*Pbca*	9.840(1)		24.416(5)		12.2489(8)		Matos Gomes et al., 1995 [16]

References

1. Canossa S., Graiff C. Role of Bis(triphenylphosphine)iminium Cation $[PNP]^+$ on the Crystal Packing of $[PNP]^+[HSeO_3]^-$ Solvate Salt. *Crystals* **2018**, *8*, 151.
2. Chen H., Gao W., Zhu M., Gao H., Xue J., Li Y. A highly selective OFF–ON fluorescent sensor for zinc in aqueous solution and living cells. *Chem. Commun.* **2010**, *46*, 8389–8391.
3. Gharzaryan V.V., Fleck M., Petrosyan A.M. New salts of amino acids with dimeric cations. *Proceedings of SPIE - International Conference on Laser Physics 2010,* **2011**, *7998*, 79980F.
4. Kamali N., O'Malley C., Mahon M.F., Erxleben A., McArdle P. Use of Sublimation Catalysis and Polycrystalline Powder Templates for Polymorph Control of Gas Phase Crystallization. *Cryst. Growth Des.* **2018**, *18*, 3510–3516.
5. Krumbe W., Haussuhl S. Structure and physical properties of orthorhombic guanidinium phthalate, $[CN_3H_6]_2C_8H_4O_4$, and guanidinium hydrogen L-aspartate, $[CN_3H_6]C_4H_6NO_4$. *Z. Kristallogr. Cryst. Mater.* **1987**, *179,* 267–279.
6. Lukevics E., Arsenyan P., Shestakova I., Domracheva I., Kanepe I., Belyakov S., Popelis J., Pudova O. Synthesis, structure and cytotoxicity of organoammonium selenites. *Appl. Organomet. Chem.* **2002**, *16*, 228–234.
7. Nemec I., Cisarova I., Micka Z. Study of the family of glycine-selenious acid addition compounds: Crystal structure of diglycine hydrogen selenite and vibrational spectra and DSC measurement of diglycine hydrogen selenite and monoglycine-selenious acid crystals. *J. Solid State Chem.* **1998**, *140*, 71–82.
8. Nemec I., Chudoba V., Havlicek D., Cisarova I., Micka Z. Preparation, Crystal Structure, Vibrational Spectra, and Thermal Behavior of N,N′-Dimethylpiperazinium(2+) Hydrogen Selenite. *J. Solid State Chem.* **2001**, *161*, 312–318.
9. Ondracek J., Walzelova M., Micka Z., Novotny J. Structure of glycine-selenious acid (1/1). *Acta Crystallogr.* **1992**, *C48*, 391–392.
10. Paixao J.A., Matos Beja A., Ramos Silva M., Alte da Veiga L., Martin-Gil J., Martin-Gil F., de Matos Gomes E. Crystal structure of betaine dihydrogen selenite, $C_5H_{13}NO_5Se$. *Z. Kristallogr. New Cryst. Struct.* **1997**, *212*, 51–52.
11. Paixao J.A., Silva M.R., Beja A.M. Crystal structure and properties of L-tryptophanium hydrogen selenite. Eusebio E. *Polyhedron* **2006**, *25*, 2021–2025.
12. Paixao J.A., Matos Beja A., Ramos Silva M., de Matos Gomes E., Martin-Gil J., Martin-Gil F.J. *N*,*N*′-diphenylguanidinium hydrogenselenite monohydrate. *Acta Crystallogr.* **1997**, *C591*, 1113–1115.
13. Ritchie L.K., Harrison W.T.A. 1-Carbamoylguanidinium hydrogenselenite. *Acta Crystallogr.* **2003**, *E59*, o1296–o1298.
14. Takouachet R., Benali-Cherif R., Bendeif E.-E., Benali-Cherif N., Pillet S., Schaniel D. Structural analysis and IR-spectroscopy of a new anilinium hydrogenselenite hybrid compound: A subtle structural phase transition. *Inorg. Chim. Acta* **2016**, *446*, 6–12.
15. Takouachet R., Benali-Cherif R., Benali-Cherif N. Cytosinium hydrogen selenite. *Acta Crystallogr.* **2014**, *E70*, o186–o187.
16. de Matos Gomes E., Matos Beja A., Paixao J.A., de Veiga L.A., Ramos Silva M., Martin-Gil J., Martin-Gil F.J. Synthesis, structure and thermal behavior of benzyltrimethylammonium trihydrogen selenite. *Z. Kristallogr. Cryst. Mater.* **1995**, *210*, 929–933.

17. de Matos Gomes E., Nogueira E., Fernandes I., Belsley M., Paixao J.A., Matos Beja A., Ramos Silva M., Martin-Gil J., Martin-Gil F., Mano J.F. Synthesis, structure, thermal and non-linear optical properties of L-argininium hydrogen selenite.*Acta Crystallogr.* **2001**, *B57*, 828–832.
18. Wang J.-J., Tessier C., Holm R.H. Analogue reaction systems of selenate reductase. *Inorg. Chem.* **2006**, *45*, 2979–2988.
